# Congenital unilateral proximal radioulnar synostosis: A surgical case report

**DOI:** 10.1097/MD.0000000000019782

**Published:** 2020-04-17

**Authors:** Yuqing Jia, Chunyuan Geng, Zikai Song, Shijie Lv, Bin Dai

**Affiliations:** a Department of Orthopedics, Jilin Province FAW General Hospital; b Department of Sports Medicine, First Bethune Hospital of Jilin University, Jilin Province; c Department of Cardiology, First Hospital of Jilin University, Changchun.

**Keywords:** bony malformation, congenital proximal radioulnar synostosis, diagnosis, pediatric orthopedics, surgical treatment

## Abstract

**Rationale:**

Congenital proximal radioulnar synostosis is a rare genetic malformation of the upper limb. This deformity, which is found mainly in preschool-aged children, has no recognized diagnosis and treatment. Current diagnostic methods cannot effectively assess both bone structure and soft tissue abnormalities, and most surgical treatments introduce complications and do not prevent recurrence. More work is needed; therefore, to address the diagnosis and treatment of this disease.

**Patient concerns:**

An 8-year-old male patient was hospitalized in our department. He reported deformity and limited motion in his right elbow for the past 2 years. He denied a traumatic or family history of bony malformation. The chief complaint at the time of the hospitalization was the limitation in forearm rotation.

**Diagnosis:**

Digital radiography of the right elbow joint showed proximal radioulnar synostosis and a valgus deformity. A 3-dimensional computed tomography scan further showed proximal ulna and radius dysplasia as well as anterior dislocation of the radius head. The patient was diagnosed with congenital right proximal radioulnar synostosis.

**Interventions:**

Surgical procedures included arthrolysis of the right proximal radioulnar joint, osteotomy of the proximal radius, internal fixation with Kirschner wires, and reconstruction of the annular ligament. The right elbow was immobilized in plaster in a flexion and supination position for 2 weeks.

**Outcomes:**

Recurrence of the right proximal radioulnar synostosis was observed during the 6-month follow-up, but the rotation function of the patient's forearm was significantly improved.

**Lessons:**

The findings from this case suggest that we should carefully monitor all patients younger than 6 years old who report long-term issues with forearm rotation. This case also highlights the need to assess soft tissue and epiphysis abnormalities in addition to bone assessments via digital radiography and 3-dimensional computed tomography. We suggest that surgery should not be performed until the proximal radius epiphysis has closed. Not all cases require surgical treatment, but when surgery is needed, a suitable method should be selected according to the individual needs of the patient. Any surgery performed should treat both the bony malformations and soft tissue abnormalities to maximize the therapeutic effect and reduce complications during and after surgery.

## 1 Introduction

Congenital proximal radioulnar synostosis is a rare orthopedic malformation. Sandifort reported the first case in 1793.^[1]^ It is an X-linked dominant disorder with a paternal pattern of inheritance, and the malformation usually involves both elbows.^[2,3]^ The exact etiology remains unclear, however, and there is no consensus regarding either its diagnosis or treatment. Herein, we report a case of congenital unilateral proximal radioulnar synostosis that was recently diagnosed and treated at our hospital. This case report is presented with consent from both the patient and his parent.

## 2 Case report

**Figure 1:**
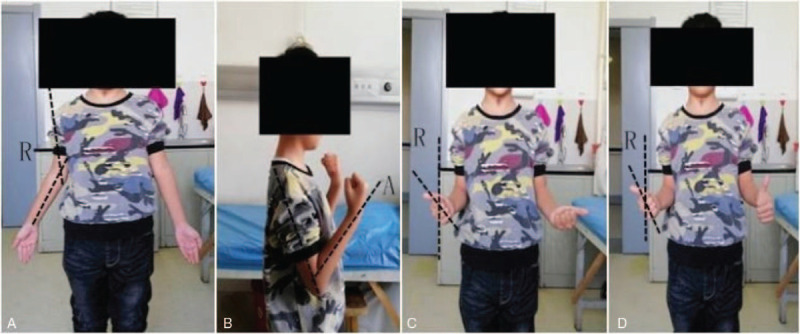
(A) Carrying angle: 25°. (B) Elbow flexion: 120°. (C) Forearm supination: 60°. (D) Forearm supination: 80°.

**Figure 2:**
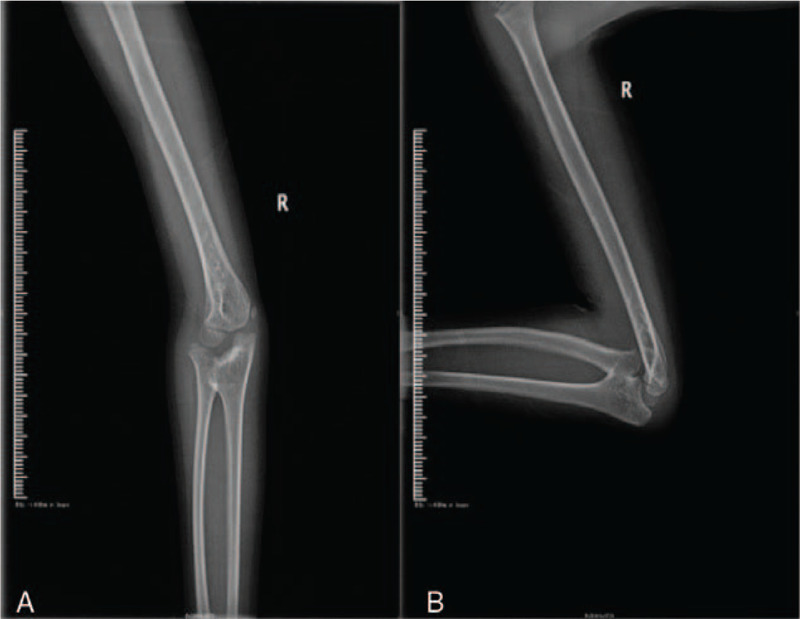
(A) Preoperative X-ray of the anteroposterior elbow. (B) Preoperative X-ray of the lateral elbow.

**Figure 3:**
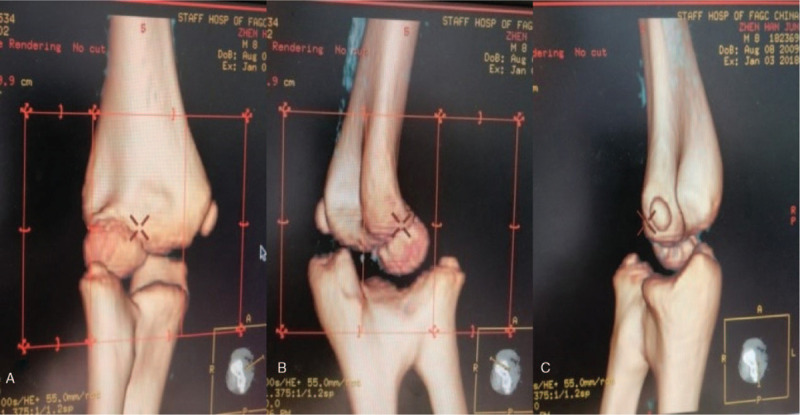
(A) Preoperative 3-dimensional computed tomography (3D CT) scan of the anteroposterior elbow. (B) Preoperative 3D CT scan of the oblique elbow. (C) Preoperative 3D CT scan of the lateral elbow.

An 8-year-old male patient who complained of deformity, pain, and limited range of motion in his right elbow for the past 2 years was hospitalized in our department in January 2018. The patient had a normal full-term delivery and did not report a traumatic or family history of malformation. Upon physical examination, the right elbow joint presented with a valgus deformity (carrying angle: 25°). No swelling was observed and no percussion pain was reported by the patient. The posterior cubital triangle of the right elbow was present. The range of elbow flexion and extension was 0° to 120°. Rotation of the right forearm was limited; the range of supination was 60° to 80° (Fig. 1). The circumference of the right mid-arm muscle was not different from that of the left arm. Myodynamia of the right arm also was normal. No other motion dysfunction was noted in the bilateral hand or wrist joints. Bilateral digital radiography (DR) of the elbows showed synostosis of the right proximal radioulnar joint as well as valgus deformity in the right elbow; no obvious abnormality was observed in the left elbow (Fig. 2). A 3-dimensional computed tomography (3D CT) scan showed dysplasia in the proximal part of the right ulna and radius as well as anterior dislocation of the radius head (Fig. 3). Based on these findings, the patient was diagnosed with congenital proximal radioulnar synostosis of the right elbow and right radial head dislocation.

**Figure 4:**
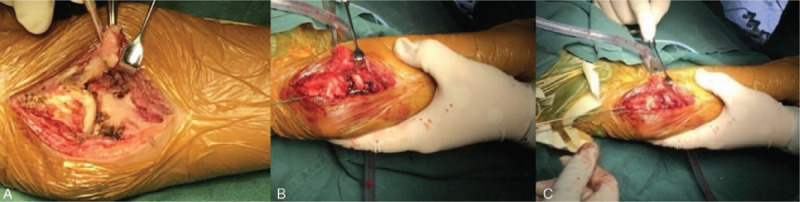
(A) Congenital proximal radioulnar synostosis and anterior dislocation of the radial head was confirmed during surgery, and the epiphysis of the radius was closed. (B) The proximal radius and ulna were separated, reduction of the radial head was performed by osteotomy, and a lateral fascia patch with blood supply was prepared to repair the annular ligament. (C) The radial head was fixed on the radial shaft through the humeral head. The elbow joint was fixed in 90° flexion, and then the annular ligament was reconstructed using the fascia patch.

The following surgical procedures were performed: arthrolysis of the right proximal radioulnar joint, proximal radius osteotomy, internal fixation with Kirschner wires, and reconstruction of the annular ligament of the radius. The posterolateral elbow approach was used. During surgery, dislocation of the radial head, synostosis of the radius and ulna, and absence of the annular ligament were confirmed. Rotation of the forearm was severely limited. The proximal fusion of the ulna and radius were separated by osteotomy after cleaning hypertrophic scar tissue under the capitulum humerus. Bone wax was applied to the surfaces of the osteotomy sites for hemostasis. Reduction of the radial head was performed via a Wedge osteotomy approximately 1.5 cm below the radial head. The radial head was fixed to the radius trunk using 2 Kirschner wires from the capitulum humerus to the radius trunk with the elbow flexed at 90° and the forearm in the supination position. A vascularized fascial patch was placed proximally between the ulna and radius to reconstruct the annular ligament (Fig. 4). The incision was closed after hemostasis and douching. The right elbow was immobilized in the flexion and supination position via a plaster cast for 2 weeks. Nonsteroidal anti-inflammatory drugs were administered to prevent heterotopic ossification.

**Figure 5:**
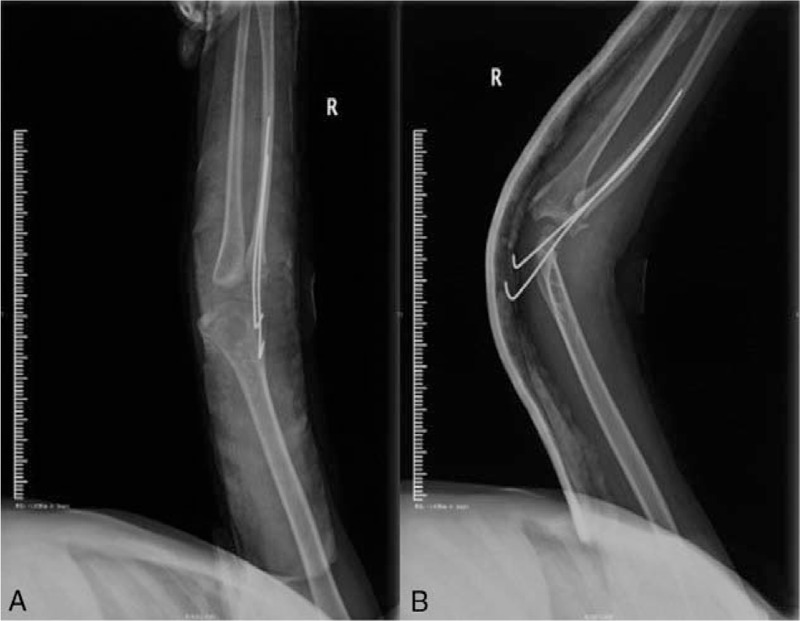
(A) Immediate postoperative X-ray of the anteroposterior elbow. (B) Immediate postoperative X-ray of the lateral elbow.

**Figure 6:**
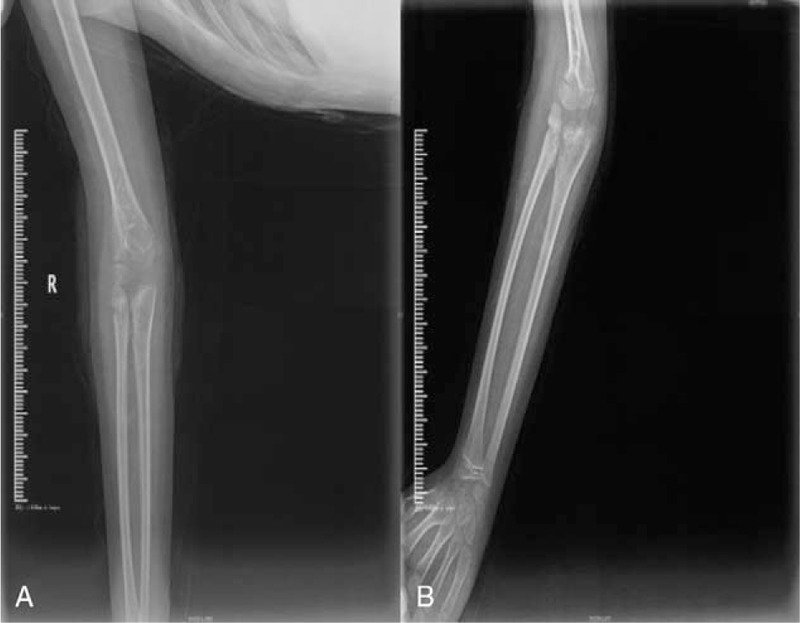
(A) X-ray of the anteroposterior elbow at the 4-wk postoperative follow-up. (B) X-ray of the lateral elbow at the 4-wk postoperative follow-up.

**Figure 7:**
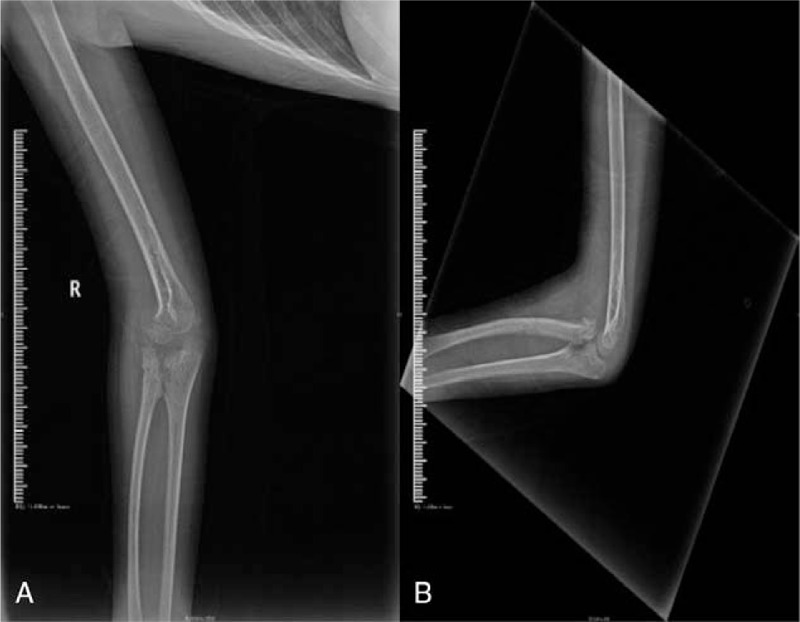
(A) X-ray of the anteroposterior elbow at the 2-mo postoperative follow-up. (B) X-ray of the lateral elbow at the 2-mo postoperative follow-up.

**Figure 8:**
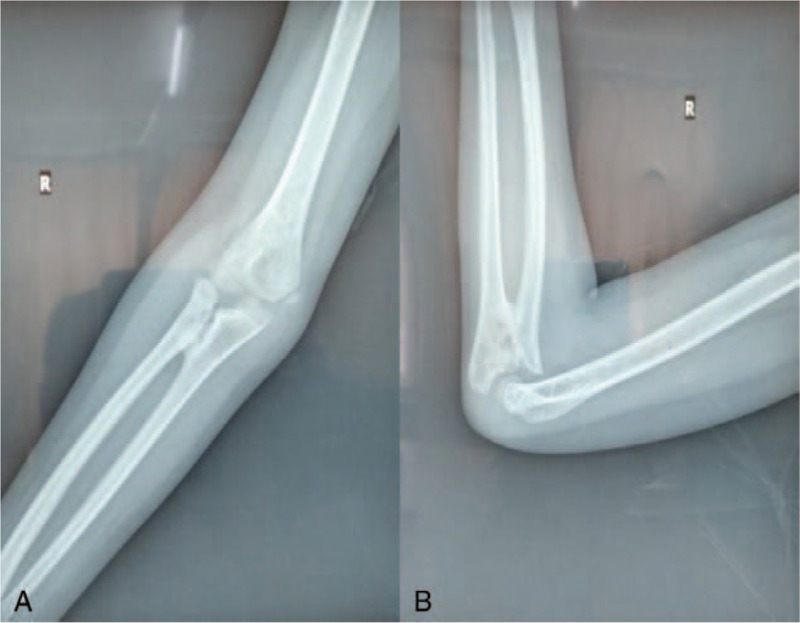
(A) X-ray of the anteroposterior elbow at the 4-mo postoperative follow-up. (B) X-ray of the lateral elbow at the 4-mo postoperative follow-up.

**Figure 9:**
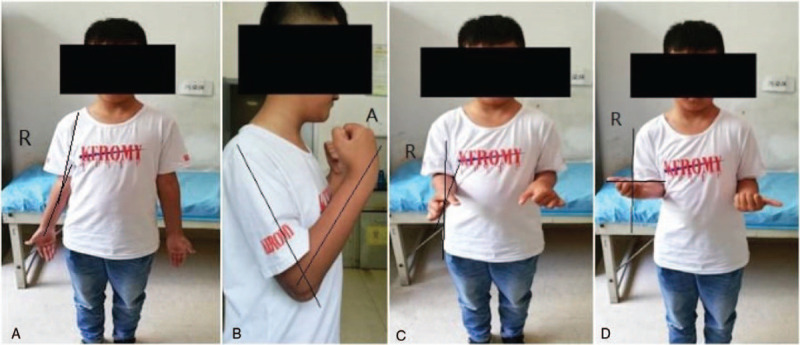
(A) Carrying angle (15°) at the 6-mo postoperative follow-up. (B) The range of flexion (130°) at the 6-mo postoperative follow-up. (C) Pronation (15°) at the 6-mo postoperative follow-up. (D) Supination (90°) at the 6-mo postoperative follow-up.

**Figure 10:**
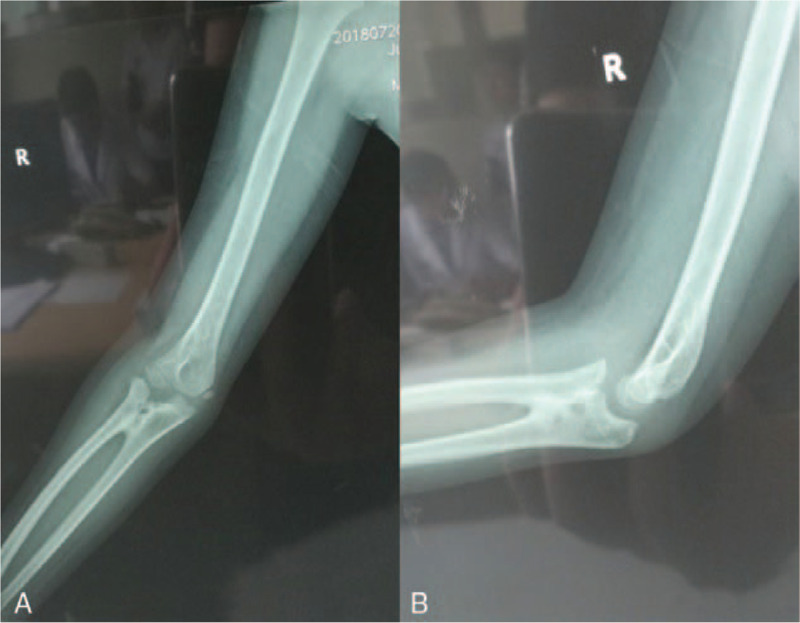
(A) X-ray of the anteroposterior elbow at the 6-mo postoperative follow-up. (B) X-ray of the lateral elbow at the 6-mo postoperative follow-up.

DR 2 weeks postoperation showed that the radial head was not dislocated and the proximal radioulnar joint had not re-fused (Fig. 5). At 4 weeks postoperation, the Kirschner wires were removed, and flexion, extension, and rotation exercises were started. DR 4 weeks postoperation showed partial union of the radial head and no obvious fusion of the proximal radioulnar joint (Fig. 6). At 2 months postoperation, the range of flexion and extension was 10° to 100°, the range of pronation was 0° to 10°, the range of supination was 0° to 60°, and the carrying angle was 15°. The 2-month postoperation DR also showed total union of the radial head with the corpus radii and no obvious fusion of the proximal radioulnar joint (Fig. 7). At 4 months postoperation, the range of flexion and extension was 0° to 130°, the range of pronation was 0° to 15°, the range of supination was 0° to 90°, and the carrying angle was 15°. Myodynamia was normal. The 4-month postoperation DR showed that the proximal radioulnar joint was in the correct position with significant osteoproliferation around the joint (Fig. 8). At 6 months postoperation, there was no significant change in function from the 4-month follow-up (Fig. 9), but the patient reported slight discomfort when the right forearm was pronated for long periods of time. The 6-month postoperation DR showed that fusion between the proximal radius and ulna had recurred (Fig. 10). The case report was waived from the Jilin Province FAW General Hospital Ethical Board, based upon their policy to review all intervention and observational study except for a case report. The patient provided informed consent for the publication of his clinical data. The presented data are anonymized and risk of identification is minimal.

## 3 Discussion

Congenital proximal radioulnar synostosis is a rare malformation of bone development characterized by the fusion of the proximal radius and ulna. This malformation usually occurs bilaterally and is diagnosed before the patient is 5 years old.^[4]^ Some studies have indicated that the incidence rate is higher in males, but a recent study found no significant difference between males and females.^[3,5,6,7]^ Although the exact etiology remains unclear, congenital proximal radioulnar synostosis has been linked to an abnormality of the distal limb on the X chromosome.^[3]^ A number of exogenous factors also may play a role, however.^[4]^ In the present case, the patient exhibited both unilateral proximal radioulnar synostosis and radial head dislocation, but reported no family or traumatic history. In addition, symptoms did not emerge until the patient was 6 years old, which is later than the typical diagnosis of this disease. Therefore, we suspected a multifactorial etiology because the patient had no other congenital anomalies.

Preoperative observations from this case suggested that the fusion site involved only the proximal epiphysis of the radius. Our theory is that because the fusion was not between 2 bony structures, the resulting cartilaginous bridge provided small extent movement when the patient rotated the forearm. In addition, we hypothesize that the rotation of the forearm was partly compensated by the distal radioulnar joint. The patient's symptoms may only have emerged after 6 years of age because this is typically when ossification of the epiphysis of the radius occurs. This ossification limits the development of the radius along the longitudinal axis of the forearm. In the presence of a malformation, the closing of the epiphysis also may have contributed to the occurrence of the valgus deformity as well as dislocation of the radial head observed in this patient. In support of this theory, preoperative DR showed that the fusion site was at the original location of the proximal radialis epiphysis. During surgery, this bony fusion was confirmed at the site of the original epiphysis of the radius. It is not surprising that the final diagnosis of congenital proximal radioulnar synostosis was initially missed in this patient, given that there was no family history or obvious relevant abnormalities. Furthermore, the cartilaginous bridge would not have been clearly visible on an X-ray. This case suggests that a patient younger than 6 years old who reports long-term issues with forearm rotation, but no family history or DR abnormality, should be considered for congenital proximal radioulnar synostosis.

Diagnosis and clinical classification of congenital proximal radioulnar synostosis usually rely on forearm rotation dysfunction and abnormalities on imaging examinations, specifically X-ray and 3D CT. Two clinical classifications were originally defined by Wilkie^[8]^: Type I, fusion of the medullary canals of the radius and ulna, in which the radius is longer and larger than the ulna; and Type II, anterior or posterior dislocation of the proximal radius with fusion to the proximal ulnar shaft. Similarly, Cleary–Omer described 4 types of synostosis based on radiography: Type I, fibrous synostosis; Type II, osseous synostosis with a normal position of the radial head; Type III, osseous synostosis with a posterior dislocation of the radial head; and Type IV, osseous synostosis with an anterior dislocation of the radial head.^[6]^ Although these fusion types can be observed clearly by DR or 3D CT, the findings from many cases suggest that synostosis often involves soft tissue abnormalities around the proximal radioulnar joint, such as contracture of the pronator and interosseous membrane and absence of the supinator muscle.^[9]^ Dynamic magnetic resonance (MR) more recently has been proposed in the diagnosis of synostosis because soft tissue abnormalities and cartilaginous connections cannot be observed on X-ray or 3D CT.^[10]^ Adoption of dynamic MR in the clinical diagnosis of synostosis has been limited; however, due to a lack of availability of equipment. The present case was diagnosed with congenital proximal radioulnar synostosis of the right elbow based on the Wilkie Type II classification and Cleary–Omer Type IV classification. Although no preoperative MR examination was conducted to assess soft tissue abnormalities in this case, surgery confirmed the absence of the annular ligament, which likely would have been observed on MR. Soft tissue abnormalities concomitant with bony malformations are common with synostosis. Given that MR is able to assess both the epiphysis and soft tissue, we suggest that preoperative MR should be considered in addition to DR and 3D CT to increase the rate of diagnosis of synostosis and better inform the treatment plan.

Clinicians do not agree on the appropriate treatment for congenital proximal radioulnar synostosis. Many clinicians recognize that bony malformations and soft tissue abnormalities usually coexist in this condition, and that surgical reconstruction of the bone alone cannot completely restore the rotation function of the forearm. In addition, there is a high postoperative rate of recurrence of the fusion.^[8]^ In contrast; however, other clinicians assert that conservative treatment will lower the patient's quality of life. In particular, as growth occurs, the bony malformation and contraction of the soft tissue will gradually increase symptoms and also will increase the risk of surgical complications. Thus, many clinicians support early intervention (including surgery) in the treatment of congenital proximal radioulnar synostosis.^[6,11]^ In our case, the rotation function of the right forearm had regressed over 2 years such that the patient was no longer able to place his elbow in the supination position. In addition, the patient reported constant pain during elbow flexion and extension. These symptoms seriously affected the quality of the patient's daily life. Surgery to separate the proximal radioulnar joint as well as to reduce the radial head and reconstruct the annular ligament relieved these symptoms and restored an appropriate forearm rotation arc that allowed for hand movement. Although the proximal radioulnar joint eventually re-fused within 6 months postoperation, the patient's forearm rotation function was improved and the patient could perform most activities of daily living. These results suggest that early-stage surgery may be the best treatment for this disease.

If surgery is the optimal treatment, then the clinician must consider the appropriate timing of surgery as well as the indications and methods to be used. Many studies suggest that early childhood is the best time for surgical treatment. For example, in a study by Murase and colleagues, all children were younger than 5 years old.^[7]^ Similarly, Fujimoto and colleagues reported that the optimal age for rotational osteotomy to treat congenital radioulnar synostosis was between 3 and 6 years old because union could be achieved without internal fixation and the radius could be sufficiently remodeled.^[2]^ Hung and colleagues reported consistent findings using derotational osteotomy, and also found better postoperative rotation function in younger versus older patients.^[11]^ There are limitations to these studies; however, including small sample sizes and lack of long-term radiographic follow-up. Our patient had surgery at 8 years old, which resulted in successful restoration of forearm rotation but did not prevent re-fusion. Based on this case, we suggest that surgery should be delayed until the proximal radius epiphysis has closed completely, which usually occurs around 7 years old, to avoid injury to the epiphysis and adverse effects on the bone union. Another advantage is that older children can more readily comply with post-operative exercise regimens to improve functional outcomes.

Clinicians do not agree on indications for surgery among patients with proximal radioulnar synostosis. Whereas some clinicians consider surgery to be necessary only when the patient's range of supination is 20° to 35°,^[9]^ others argue that surgery should be performed whenever the patient's daily life or sporting activities are negatively impacted.^[12]^ In recent years, a group of surgeons proposed that surgical treatment should be indicated under 2 circumstances:

(1) When the forearm is locked in a position of hyperpronation (ie, pronation >90°).
(2) When there is bilateral involvement.^[13]^

In reality, there are many factors that may influence the decision to pursue surgical treatment, such as whether the patient has a unilateral or bilateral malformation, is right- or left-hand dominant, uses knives or chopsticks, or plays or is learning a musical instrument. The indication for surgery also may depend on whether the shoulder and wrist joints can compensate for the forearm's rotation function or whether other deformities are present. Surgery should be performed; therefore, to relieve pain and restore whatever forearm rotation function is suitable to the individual patient's needs.

In general, we suggest the following guidelines should be used to determine whether surgery is indicated:

(1) The dominant-side forearm is locked in 0° to 20° pronation or 60° to 90° degrees supination.
(2) The dominant-side forearm is locked in 20° to 60° pronation or 20° to 60° degrees supination and the dysfunction cannot be compensated by the ipsilateral shoulder or wrist.
(3) There is bilateral forearm rotation dysfunction that cannot be compensated by the shoulder or wrist and has a significant effect on the patient's quality of daily life.
(4) The patient has any of the above in combination with dislocation of the radial head and significant pain.

There is no gold-standard method for treating congenital proximal radioulnar synostosis. Radioulnar rotation osteotomy is common, but requires a long period of immobilization of the forearm post-operation and is associated with an increased risk of delayed union, nerve injury, and ischemic muscle contracture.^[9,14,15]^ Hung and colleagues reported on the use of derotational osteotomy in 34 cases, all of which experienced significant improvement in rotation function with no complications. This procedure is complicated to perform; however, which limits its widespread use. In addition, like radioulnar rotation osteotomy, recovery requires a long period of immobilization of the forearm.^[11]^ Others have reported the use of vascularized fascia-adipose layer transplantation, in which the fusion site is separated and the radial head is wrapped with pedicled fascia. While this treatment has been reported to improve the rotation function of the forearm and reduce the rate of recurrence, a few cases developed transient posterior interosseous nerve palsy postoperation.^[16,17]^ In addition, Kanaya and colleagues, who performed free vascularized fascio-fat grafting in 7 cases, reported recurrence of synostosis in 3 of the cases.^[18]^

In our case, we separated the ossified proximal radioulnar joint, performed a Wedge osteotomy, and fixed the caput of the radius to the radialis trunk with 2 Kirschner wires. We then used a lateral vascularized fascial patch to reconstruct the annular ligament. The patient's forearm was immobilized for 2 weeks postoperation. To reduce the possibility of re-fusion, we smeared bone wax on the radioulnar surfaces during surgery and administered indomethacin postoperation. We simplified the osteotomy to minimize the risk of delayed union, nerve injury, and ischemic muscle contracture. We chose a fixation approach that considered the patient's trait of growth and reduced the immobilization time. Despite the use of the vascularized fascial patch, bone wax, and indomethacin to prevent recurrence; however, the proximal radioulnar joint was re-fused at the 6-month follow-up. Based on our findings, we propose the following guidelines to improve postoperative outcomes:

(1) Correct any radial head dislocation.
(2) Choose an appropriate fixation, if osteotomy is needed, to reduce the immobilization time and prevent delayed union or nonunion.
(3) Correct as much of the supination deformity as possible, and maintain the forearm in a neutral position (eg, using internal or external fixation) during and after the operation.
(4) Protect the ulna and radius epiphysis from injury in cases when the surgery occurs before the proximal radius epiphysis has closed.
(5) Repair soft tissue abnormalities around the proximal radioulnar joint to reinforce the stability of the bone structure.
(6) Inform the patient and their parents before the operation that the prognosis may be unsatisfactory due to the high rate of recurrence.

In conclusion, congenital proximal radioulnar synostosis is a rare genetic disease. There is no consensus regarding its diagnosis or treatment. Soft tissue abnormalities involving the epiphysis usually accompany the bony malformation, indicating that an assessment of both the soft tissue and epiphysis is necessary. The findings from our case suggest that we should consider congenital proximal radioulnar synostosis in patients younger than 6 years old if they report long-term forearm rotation dysfunction and no prior family history, as early-stage surgery may benefit these patients. The surgical methods performed should be selected according to the individual needs of the patient, and should treat both the bony malformation and the soft tissue abnormality with an appropriate fixation to reduce complications and maximize the therapeutic effect. We believe that treatment of congenital proximal radioulnar synostosis will continue to be improved as orthopedic technology continues to develop.

## Acknowledgments

The authors thank the patient and his family for their participation and the BioScience Writers for their editing.

## Author contributions

**Data curation:** Chunyuan Geng.

**Supervision:** Bin Dai.

**Writing – original draft:** Yuqing Jia.

**Writing – review & editing:** Zikai Song, Shijie Lv.
